# Rat Hepatitis E Virus (*Rocahepevirus ratti*): A Systematic Review of Its Presence in Water, Food-Related Matrices, and Potential Risks to Human Health

**DOI:** 10.3390/foods14142533

**Published:** 2025-07-19

**Authors:** Sérgio Santos-Silva, Helena M. R. Gonçalves, Wim H. M. Van der Poel, Maria S. J. Nascimento, João R. Mesquita

**Affiliations:** 1 School of Medicine and Biomedical Sciences (ICBAS), University of Porto, 4050-313 Porto, Portugal; 2 LAQV, REQUIMTE, Department of Chemistry and Biochemistry, Faculty of Sciences, University of Porto, 4200-072 Porto, Portugal; 3 Infectious Diseases Epidemiology, Wageningen University, 6708 PB Wageningen, The Netherlands; 4 Department Virology & Molecular Biology, Wageningen Bioveterinary Research, 8200 AB Lelystad, The Netherlands; 5 Faculty of Pharmacy, University of Porto, 4050-313 Porto, Portugal; 6 Centre for the Study of Animal Science (CECA), Institute for Science, Technology and Environment (ICETA), University of Porto, 4051-401 Porto, Portugal; 7 Associate Laboratory for Animal and Veterinary Science (AL4AnimalS), 1300-477 Lisboa, Portugal

**Keywords:** HEV, *Rocahepevirus ratti*, One Health, systematic review

## Abstract

Rat hepatitis E virus (rat HEV) is an emerging zoonotic virus detected in rodents worldwide, with increasing evidence of presence in environmental sources such as surface water, wastewater and bivalves. This systematic review compiles and analyzes all the published research on rat HEV contamination in these matrices, as well as its implications for human health. A comprehensive literature search was conducted using databases such as PubMed, Scopus, Web of Science, and Mendeley, including studies published up until 27 May 2025. Studies were included if they evaluated rat HEV in water- or food-related matrices using molecular detection. The risk of bias was not assessed. The certainty of evidence was not formally evaluated. Limitations include reliance on PCR methods without infectivity confirmation. Following PRISMA inclusion and exclusion criteria, eight eligible studies were analyzed. The results show high detection rates of rat HEV RNA in influent wastewater samples from several high-income European countries, namely Sweden, France, Italy, Spain and Portugal. Lower detection rates were found in effluent wastewater and surface waters in Sweden. In bivalve mollusks sampled in Brazil, rat HEV RNA was detected in 2.2% of samples. These findings show the widespread environmental presence of rat HEV, particularly in urban wastewater systems. While human infections by rat HEV have been documented, the true extent of rat HEV zoonotic potential remains unclear. Given the risks associated with this environmental rat HEV contamination, enhanced surveillance, standardized detection methods, and targeted monitoring programs in food production and water management systems are essential to mitigate potential public health threats. Establishing such programs will be crucial for understanding the impact of rat HEV on human health.

## 1. Introduction

Hepatitis E virus, a small, non-enveloped RNA virus with a positive-sense single-stranded genome, classified under the family *Hepeviridae*, subfamily *Orthohepevirinae*, species *Paslahepevirus balayani* (HEV), has been, for some time, the primary research focus of human hepatitis E infections. Up until recently, it was thought to be the only species within the family *Hepeviridae*, known to infect humans, with genotypes HEV-3, HEV-4, and HEV-7 demonstratingzoonotic potential, infecting humans and various animal hosts such as swine, wild boars, rabbits, and camels [1].

Recently, with the detection of rodent HEV in humans, *Rocahepevirus ratti*, another species within the family *Hepeviridae*, has been implicated in human infections [2,3,4]. The first known human case was reported in 2018 when rat HEV was detected in a liver transplant recipient in Hong Kong, challenging prior assumptions about the host range of the family *Hepeviridae* [4]. Since then, nearly 30 human cases of acute and chronic hepatitis linked to rat HEV have been reported across multiple continents, including Asia, Europe, and North America [3,5,6,7,8]. These cases have raised concerns about the zoonotic potential of rat HEV, particularly given its ability to infect both immunocompromised and immunocompetent individuals [5,8].

Despite growing recognition of rat HEV as an emerging pathogen, key questions persist regarding its transmission dynamics, reservoirs, and impact on public health. Unlike *Paslahepevirus balayani*, with a well-documented transmission through foodborne and fecal-oral routes, the pathways through which rat HEV infects humans remain unclear. To date, only one case has been directly linked to rodent contact [8], suggesting the potential involvement of alternative hosts or foodborne sources [9].

This systematic review compiles and analyzes the current state of *Rocahepevirus ratti* research, with a particular focus on its presence in water- and food-related matrices and its implications for human health. Given the potential for indirect zoonotic transmission and the increasing detection of rat HEV in diverse environmental contexts, a One Health framework is essential to understand the interconnection between humans, animals, and their shared environments.

## 2. Materials and Methods

A comprehensive literature search was conducted using the electronic databases Mendeley, PubMed, Scopus, and Web of Science, considering studies published up until 27 May 2025. This systematic review adhered to the Preferred Reporting Items for Systematic Reviews and Meta-Analyses (PRISMA) guidelines [10], ensuring the inclusion of only published, peer-reviewed, and indexed studies. To maintain linguistic consistency, only articles written in English were considered.

The search strategy involved using specific keywords such as “rat hepatitis E virus”, “*Rocahepevirus ratti*”, and “*Orthohepevirus C*” across the selected databases. Search terms included combinations of “rat hepatitis E virus”, “*Rocahepevirus ratti*”, “*Orthohepevirus C*”, “wastewater”, “surface water”, and “bivalves”. The inclusion of “bivalves” as a keyword was deliberate, reflecting their recognized role as a food matrix frequently implicated in the transmission of enteric viruses to humans. Boolean operators and MeSH terms were used as appropriate. Articles that did not focus on the detection, epidemiology, or dynamics of rat HEV in waters and food were excluded based on a preliminary screening of titles and abstracts. In cases where relevance was unclear, full-text articles were reviewed for clarification. No formal risk of bias assessment was conducted for the included studies, as most were environmental surveillance studies without outcome comparators.

Data extracted included study location, sampling date, matrix type (influent/effluent wastewater, surface water, bivalves), detection method, genomic target, HEV and rat HEV prevalence, and genotypes. No assumptions were made regarding missing or unclear data.

The certainty of the evidence was not formally assessed using GRADE or similar frameworks due to the descriptive nature of the included studies and the lack of effect measures.

**Figure 1:**
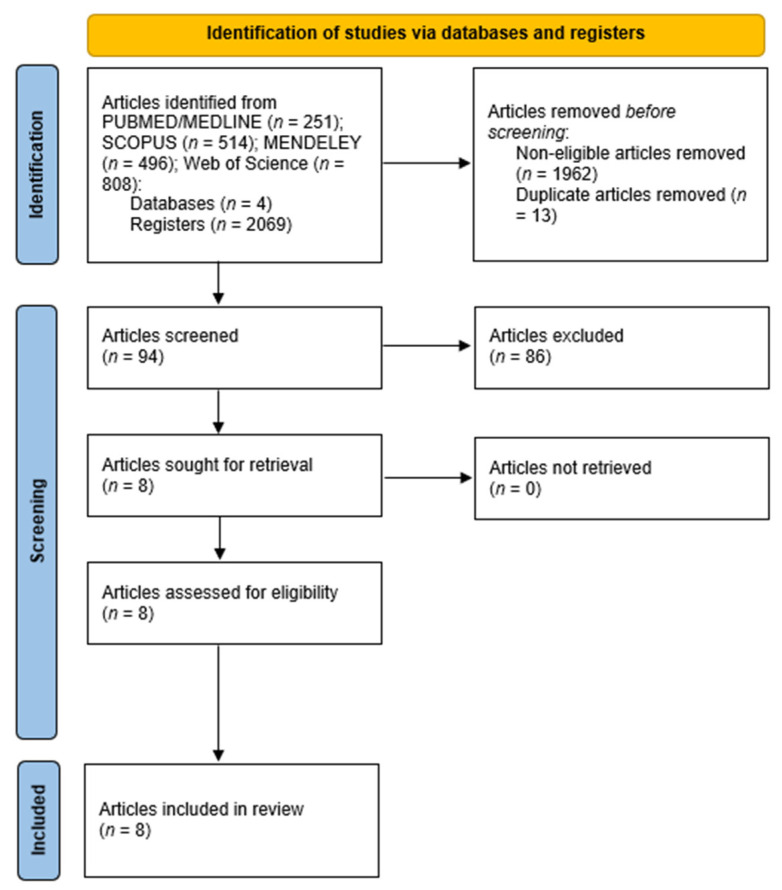
PRISMA flow diagram illustrating the stages of record selection and detailing the inclusion and exclusion criteria, along with their justifications.

Two independent researchers (S.S.-S. and J.R.M.) conducted the database screening and extracted relevant data. After applying the inclusion and exclusion criteria, eight articles were identified as potentially suitable for the systematic review. Following a full-text assessment, all eight articles met the eligibility criteria. The study selection process is detailed in Figure 1.

## 3. Results

A total of 2069 articles were identified through systematic searches across four electronic databases: PUBMED/MEDLINE (*n* = 251), SCOPUS (*n* = 514), Mendeley (*n* = 496), and Web of Science (*n* = 808). After removing duplicates and non-eligible articles, 94 articles were selected based on titles and abstracts for further screening. Of these, 86 articles were excluded for not meeting the predefined inclusion criteria. The full texts of the remaining eight articles were retrieved and assessed in detail. Following this evaluation, all eight articles met the eligibility criteria and were included in the final analysis. Table 1 summarizes the main characteristics of each study, including the country of origin, sample type, detection method, and key findings.

**Figure 2:**
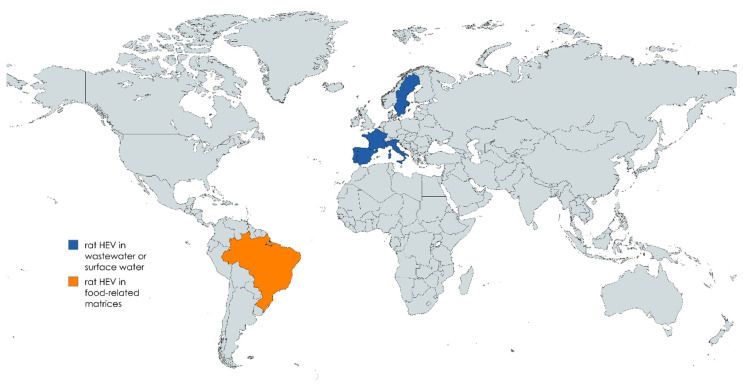
Countries where rat HEV has been detected in wastewater and surface water (shown in blue) and in food-related matrices (shown in orange).

Eight studies assessed the presence of rat HEV and HEV in environmental and food-related matrices across Europe and Brazil (Figure 2), employing primarily RT-qPCR assays targeting the ORF1 region, with some studies incorporating metagenomic sequencing approaches.

The majority of studies focused on wastewater samples. Influent wastewater from sewage treatment plants in Sweden, Italy, France, Spain and Portugal showed rat HEV detection rates ranging from 24.32% to 100% [11,12,13,14,15,17,18]. In these same samples, HEV RNA was also detected, although with considerable variation. In Spain, only 0.9% and 14.29% of samples tested positive for HEV [15,18]. In contrast, 86% and 100% of influent samples from Sweden were positive for HEV strains [13,17], while 4.5% of Italian samples harbored HEV RNA [12]. In France, HEV RNA was detected in 91% of influent wastewater samples [14].

Effluent wastewater from Swedish treatment plants showed a lower positivity rate for rat HEV, with only 18% of samples testing positive [11]. Nevertheless, HEV RNA was still detected in 63.6% of treated wastewater samples observed in that study. Viral loads followed seasonal patterns and were found to precede reported clinical cases by two to four weeks, despite wastewater treatment reducing viral concentrations [11].

Surface water samples were also included in the surveillance. In France, surface river water samples showed 100% positivity for both rat HEV and HEV [14]. Conversely, lake water samples collected in Sweden during the same period tested negative for rat HEV [13].

Wastewater from swine slaughterhouses was also analyzed. Rat HEV was not detected in any samples [14], whereas HEV RNA was identified in 100% of the samples, again with genotypes 3c and 3m reported.

One study conducted in Brazil examined bivalve mollusks (mussels and oysters) intended for human consumption [16]. Among the 89 shellfish samples tested, 2.2% were found to be contaminated with rat HEV, indicating a potential route for foodborne transmission via fecal contamination in aquatic environments. No HEV RNA was detected in any of the bivalves samples tested.

Due to the observational and surveillance nature of the included studies, risk of bias and certainty assessments were not performed. This represents a gap for future systematic reviews.

## 4. Discussion

In recent years, the detection of rat HEV in both environmental and clinical contexts has gained increasing attention, prompting critical questions about its potential for zoonotic transmission and its significance for public health [5,9]. While HEV genotypes, such as genotype 3, have been shown to be transmitted through contaminated water [19], with swine and wild boar identified as the main reservoirs for human infections, the detection of rat HEV in surface water, wastewater and bivalves suggests an additional, previously overlooked possible transmission route. The primary routes of transmission remain under investigation, but drinking water contamination, food products exposition to rodent activity, and potential cross-contamination in agricultural and urban settings are key concerns.

The eight studies analyzed in this systematic review reflect a growing interest in the detection of rat HEV in water and food-related matrices, highlighting the expanding ecological presence of rat HEV and the implications for human exposure. Although only eight studies met the inclusion criteria, they represent the total body of peer-reviewed literature currently available on rat HEV in environmental and food-related matrices. This reflects both the novelty of the topic and the growing interest in this emerging zoonotic virus. A consistent finding across these studies was the high detection rates of rat HEV RNA in urban wastewater networks of high-income European countries, such as Sweden, France, Italy, Spain and Portugal [11,12,13,14,15,18]. These findings point to a widespread environmental circulation of rat HEV, likely originating from urban rodent populations.

The Swedish study reported that 20 out of 21 rat HEV sequences detected in influent wastewater in Gothenburg clustered within the genotype C1 [13]. In the Nancy region, the first study in France to report the detection of rat HEV in wastewater further confirmed the presence of the virus in densely populated, rodent-rich environments, but genotype was not determined [14]. In the south of Italy, in the city of Abbruzo, a 43.9% detection rate of rat HEV genotype C1 was shown [12]. Curiously, the study conducted in Spain found a correlation between wastewater levels of HEV/rat HEV and human cases of hepatitis E, highlighting the potential of wastewater surveillance as a public health tool [15]. In a study conducted in northern Portugal and Spain, HEV was nearly absent in influent wastewater and swine fecal samples, while high rat HEV levels in the influent wastewater suggested potential urban transmission risks and highlight the need for better zoonotic virus surveillance [18]. However, comprehensive data on the specific contribution of rat HEV to human infections remain limited, as most clinical cases are associated with HEV genotypes 3 and 4. Nonetheless, in Hong Kong rat HEV has been shown to account for approximately 10% of all hepatitis E cases and around 40% of chronic hepatitis E infections in immunocompromised individuals [8,20]. Moreover, human infections with rat HEV have been spatially linked to epizootics in local rat populations [20]. The detection of rat HEV in wastewater highlights the potential for indirect human exposure through environmental contamination, especially in areas with inadequate wastewater treatment infrastructure, dense rodent populations, or poor sanitation, emphasizing the risk of its entry into human water supply chains. There are notable findings regarding the detection of rat HEV across different sampling points within wastewater treatment systems, including influent, effluent, and surface water receiving treated wastewater. Some studies have reported high detection rates in influent wastewater samples [13,14,18], reflecting the frequent presence of rat HEV RNA entering wastewater treatment plants, likely originating from infected urban rodent populations. Nevertheless, detection in influent wastewater samples alone does not necessarily imply a direct risk to public health, as viral particles may be substantially reduced or eliminated during wastewater treatment processes. However, the detection of rat HEV RNA in effluent wastewater samples (treated wastewater) suggests that current treatment processes may not completely eliminate viral RNA [11,14]. Additionally, the presence of rat HEV in surface waters downstream of wastewater discharge points raises further concerns about its environmental persistence and potential spread [14]. Given that surface waters are frequently used for recreational activities like swimming and water sports, the detection of rat HEV in both surface waters and treated effluent underscores a potential route for indirect human exposure, emphasizing the need for improved monitoring and treatment strategies to mitigate public health risks.

Beyond wastewater samples, the detection of rat HEV RNA in bivalve mollusks intended for human consumption [16] raises concerns about potential health risks, particularly given the common consumption of raw or undercooked shellfish in many regions. This finding aligns with previous concerns regarding the transmission of other enteric viruses, such as norovirus, HEV and hepatitis A virus [21,22,23], and supports prior studies demonstrating the occurrence of enteric viruses in shellfish [24,25].

Considering these potential transmission routes, particularly via effluent wastewater and surface water or contaminated food sources, it is important to evaluate which populations may be at greater risk of exposure. Individuals living in urban centers with high rodent activity, communities with poor sanitation, and immunocompromised individuals could be more vulnerable to the infection of rat HEV. While direct evidence of transmission remains limited, this ecological pattern suggests a need for risk stratification and prioritization of surveillance in higher-risk settings. Integrating environmental, clinical, and demographic data would be instrumental in assessing the actual risk to public health.

Although not considered in this systematic review due to not being detected in pork food products, rat HEV has been detected in stools of swine, suggesting that pigs may be alternative reservoir hosts for this virus, and raising questions about possible viral replication in pig tissues [26]. Interestingly, another study successfully demonstrated the susceptibility of swine to rat HEV, suggesting that pigs may act as potential intermediate or amplification hosts, thereby playing a role in the transmission cycle of this emerging pathogen [27]. Surveillance programs targeting food sources, such as shellfish and pork, along with rigorous testing, are essential to preventing potential foodborne transmission linked to this emerging zoonotic virus. While the possibility of rat HEV transmission through food remains speculative and requires further evidence, the evidence of rat HEV in bivalve mollusks intended for human consumption emphasizes the need for research into foodborne transmission pathways. Future studies should focus on confirming whether rat HEV can infect pigs and contaminate other food-related matrices, potentially entering the human food chain, as well as developing effective monitoring strategies to assess the risk associated with pork consumption.

Even though the evidence of the detection of rat HEV in environment and bivalves is relevant, a key limitation in the current body of evidence is the lack of confirmation regarding viral infectivity and zoonotic potential. Most studies, including those analyzed in this systematic review, relied on PCR-based methods that detect viral RNA but have the disadvantage of not providing information about the virus infectivity. Furthermore, despite sporadic reports of rat HEV RNA in human clinical samples, genetic divergence between human and environmental suggests limited or yet uncharacterized crossover events [13]. Specific limitations for this review are the lack of formal risk of bias assessment and absence of certainty grading.

Additionally, a recent study has reported the detection of rat HEV in companion animals, including cats and dogs, further broadening the potential host range of this virus and highlighting the importance of a One Health approach to surveillance [28]. These findings raise concerns about the role of domestic animals in the ecology and transmission dynamics of rat HEV, particularly in urban settings where close human–animal interactions are frequent.

The recurrent identification of rat HEV in multiple countries, provides robust evidence that rat HEV is more prevalent and geographically widespread than previously recognized. To improve data comparability and global surveillance, it is important to standardize detection protocols, including sample concentration, extraction methods, and genomic target regions. Uniform methodologies would facilitate meta-analyses and enable better risk assessment across different regions.

While direct human–rodent interactions may be less sustained in contemporary settings, urbanization, environmental changes, and shifts in ecological dynamics may facilitate novel interfaces between human populations, rodents, and their surrounding environments. In light of these factors, a One Health approach is critical for a comprehensive understanding of the emergence and transmission dynamics of rat HEV.

## 5. Conclusions

In conclusion, the presence of rat HEV in surface water, wastewater and food-related matrices, such as bivalve mollusks, reflects a growing environmental burden with potential public health implications. Though definitive waterborne transmission of rat HEV has yet to be proven, these findings warrant further investigation into exposure risks, viral infectivity, and possible waterborne transmission of rat HEV. As with prior emerging pathogens, early recognition and systematic surveillance are key to mitigating future risks. More integrative, One Health oriented studies are needed to assess the transmission dynamics between rodents, the environment, and human populations, particularly in rural settings where these interactions are most frequent. Although the number of available studies on this topic remains limited, the findings consistently indicate that rat HEV is present in environmental and food-related matrices, supporting its relevance as an emerging public health concern and highlighting the need for further research and surveillance. Ongoing environmental monitoring and cross-disciplinary collaboration will be key to understanding whether rat HEV poses a sporadic or emerging threat to human health.

## Figures and Tables

**Table 1:** Summary of studies reporting the presence of rat HEV and HEV in water and food-related matrices.

Sampling Country (City)	Sampling Date	Sample Type	Rat HEV Detection Assay	Rat HEV Target Region	Number of Rat HEV RNA Positive/Total (%)	Rat HEV Genotype	HEV Detection Assay	HEV Target Region	Number of HEV RNA Positive/Total (%)	HEV Genotype/Subtype	Main Findings	Reference
Sweden (Gothenburg)	2016–2017	Influent and Effluent wastewater	RT-qPCR	ORF1	Influent wastewater (NA), Effluent wastewater 2/11 (18.18%)	ND	RT-qPCR	NA	Influent wastewater (NA), Effluent wastewaters 5/11 (63.64%)	3c, 3f, 3i	HEV and rat HEV were both detected in effluent wastewaters. Peaks in virus concentration in wastewater preceded clinical cases by 2–4 weeks. Despite wastewater treatment significantly reducing viral loads, HEV was not completely eliminated and substantial amounts of both human and rat HEV strains were discharged into the environment via effluent.	[11]
Italy (Abruzzo)	2019–2022	Influent Wastewater	pan-hepevirus heminested RT-PCR	ORF1	68/155 (43.9%)	C1	pan-hepevirus heminested RT-PCR	ORF1	7/155 (4.52%)	3	Rat HEV was a significant component of the wastewater microbiota	[12]
Sweden (Gothenburg)	2023	Influent wastewater, Surface water	RT-qPCR	ORF1	Influent wastewater 50/51 (98%), Surface (lake) Water 0/16 (0%)	C1	RT-qPCR	NA	Influent wastewater 41/51 (86%), Surface (lake) Water 0/16 (0%)	3b, 3c, 3i	High prevalence of HEV and rat HEV, with dominant HEV-3c/i subtypes. Previously unreported HEV3b and unclassified HEV strains detected in wastewater. Distinct rat HEV clades found in wastewater that had not been previously seen in humans	[13]
France (Nancy)	2023	Influent wastewater, Slaughterhouse wastewater, Surface water	RT-qPCR	ORF1	Influent wastewater 11/11 (100%), Slaughterhouse wastewater 0/14 (0%), Surface (river) water 4/4 (100%)	ND	RT-qPCR	NA	Influent wastewater 10/11 (91%), Slaughterhouse wastewater 14/14 (100%), Surface (river) water 4/4 (4%)	3c, 3m	High concentrations of *Rocahepevirus* (up to 40-fold higher than that of *Paslahepevirus*) were detected in urban wastewater and rivers, suggesting potential for human transmission, though no cases were found in local hepatitis patients, highlighting the need for improved detection methods	[14]
Spain (Cordoba)	2021–2023	Influent wastewater	RT-qPCR	ORF1	100/106 (94.3%)	C1	RT-qPCR	ORF3	1/106 (0.9%)	3f	First study in Spain to assess rat HEV in wastewater alongside clinical data, found no direct correlation with clinical cases	[15]
Brazil (São Luís)	2023–2024	Bivalves (mussels and oysters)	pan-hepevirus heminested RT-PCR	ORF1	2/89 (2.2%)	C1	RT-qPCR	Overlapping region of ORF-2/ORF-3	0/89 (0%)	ND	First report of rat HEV in bivalve mollusks; may prompt further research on its variability, distribution, host range, and potential transmission via contaminated shellfish	[16]
Sweden (Gothenburg)	2016–2018	Influent wastewater	nested RT-PCR	ORF1	9/37 (24.32%)	ND	RT-qPCR	ORF3	37/37 (100%)	3c	The study found distinct HEV-3 subtype distributions in Sweden, with subtype 3c dominant in water and humans, and 3f in pigs and wild boars, indicating multiple transmission routes	[17]
Portugal and Spain (Porto and Santiago de Compostela)	2020–2022	Influent wastewater	RT-qPCR	ORF1	39/44 (88.64%)	C1	RT-qPCR	ORF3	3/44 (6.82%)	ND	HEV was nearly absent in influent wastewater and swine fecal samples, while high rat HEV levels in the influent wastewater suggest potential urban transmission risks and highlight the need for better zoonotic virus surveillance	[18]

RT-qPCR—Reverse Transcription Quantitative Polymerase Chain Reaction; RT-PCR—Reverse Transcription Polymerase Chain Reaction; ORF—Open Reading Frame; ND—Not Determined; NA—Not Available.

## Data Availability

No new data were created or analyzed in this study. Data sharing is not applicable to this article.

## Abbreviations

The following abbreviations are used in this manuscript:

HEV	Hepatitis E Virus
rat HEV	Rat Hepatitis E Virus
RT-qPCR	Reverse Transcription Quantitative Polymerase Chain Reaction
RT-PCR	Reverse Transcription Polymerase Chain Reaction
ORF	Open Reading Frame
ND	Not Determined
NA	Not Available
PRISMA	Preferred Reporting Items for Systematic Reviews and Meta-Analyses
RNA	Ribonucleic Acid
